# Illegitimate bodies? Turner syndrome and the silent interplay of age, gender, and generational positions

**DOI:** 10.3389/fsoc.2023.1084707

**Published:** 2023-03-08

**Authors:** Nicoletta Diasio

**Affiliations:** Faculty of Social Sciences, University of Strasbourg, Laboratoire interdisciplinaire en ètudes culturelles (UMR CNRS 7069), Institut Universitaire de France, Strasbourg, France

**Keywords:** body, time, age, gender, generation, Turner syndrome, developmental thinking, medical anthropology

## Abstract

This paper focuses on the strength of social norms that define the right development of the body in time. It also analyzes how the social positions of age, gender and generation intertwine in the definition of such a legitimate body. The starting point is anthropological research carried out in France between 2018 and 2020 among girls and women affected by Turner syndrome, a rare genetic condition causing small stature, ovarian insufficiency, a delay or absence of puberty, and infertility. We first explore how measuring the body has become central in the social construction of the concept of age-appropriateness. We then present four women' narratives, which express various forms of desynchronization: the gap between physical appearance, chronological age and age status; the cleft between the physical development induced by hormone therapy and being in a particular stage in life; the difference between chronological and reproductive age; and lastly, the trouble in a generational position related to infertility. For women suffering from this genetic condition, the gap between bodies, time and social statuses associated with age, gender and generation, may engender a feeling of “being out of place.” The alignment of body and time is then one of the bastions of essentialization and naturalization. Finally, we stress the complex interplay of bodily and social markers of age and gender, and their role in social relations as both a resource and a constraint. Thus, while the syndrome may cause distress and sometimes a lack of legitimacy, it also leads to a critical re-examination of hegemonic models of womanhood and their intersection with age positions.

## Introduction

Anthropology has for long explored how “the body is used to think time, and how it also, in turn, is used to set the clock of the temporal thresholds which society invents” (Julien and Voléry,  2019: p. 7). Since the beginning of the 20th century, ethnologists have demonstrated how societies carefully organize the coupling of bodies and times in defining age and social transitions. More specifically, these passages—births, deaths, the end of childhood, the beginning of adulthood, the start and the end of procreative or sexual periods—all constitute opportunities to re-establish individuals and groups in time: in an age, in a life's stage, and in a generational position. Thus, despite the academic and social discourse about the dynamic character of the life course (Elder et al., 2003), and despite the reconfiguration of the categories of age (Blatterer, 2007a,b), and gender performativity (Butler, 2004), the social norms that establish a conformity between bodily transformations and stages in life have not disappeared: they mutate and reemerge in different forms. Expert knowledge is still home to norms that codify the link between body and time. The “surgery of age” (Moulinié, 1998) aligns the human body with standards of development or shields it from the ravages of time (Vincent, 2006; Dalgalarrondo and Hauray, 2015). Through hormonal corrections, a biological materiality is adjusted to social ambitions of self-optimization, to gender social identities, and to age status (Oudshoorn, 1994; Conrad and Potter, 2004; Toër-Fabre and Levy, 2007; Fishman et al., 2010).

This paper aims to show how age categories, gender norms and generational position intertwine in the definition of a “legitimate body” (Boni-Legoff, 2016). According to Isabelle Boni-Legoff, this legitimacy is based on the opposition which hierarchizes bodies between masculine (legitimate) and feminine (illegitimate). She also underlines the importance of the triad “gender, class, race” in defining the contours of conformity to hegemonic norms. This approach, however, underestimates two aspects. The first is the place of bodily materiality itself, which is inseparable from its discursive setting and foundational to the experience that the actors have of this legitimacy.^1^ The second is the importance of the age position in the social structure and in the process of constructing personhood. The under-estimation of age categories, compared to the value given to gender, ethnicity, race and class, has been stressed by other authors, particularly those working in fields where age-related asymmetries are more apparent, such as childhood (James and Prout, 1997) or aging (Rennes, 2020).^2^

Finally, the position in the generational order as a central dimension of the “legitimate body” is relatively absent from the scientific debate.^3^ Gender distinction—whether it is assigned, negotiated or reformulated over time—age and generation constitute the very nexus between the body, the short time spans of the individual life, and the longer times which are perpetuated through the lines of descent. “Whether it is seen as the biographical time that brings an individual from conception to birth and then to death, or the historical time that distinguishes the past, the present and the future of a society, or the socio-cosmic time spanning cycles of metamorphosis and the regeneration of beings, the masculine/feminine distinction is continually deployed and changed, constructed and altered” (Théry, 2008: p. 31).

The starting point of this article is a research study on the experience of women affected by Turner syndrome, a genetic condition causing slowed growth and variations in physical and sexual development. The syndrome upends the apparent concordance between the time of growth, the social age, the expressions of conventional femininity that emerge over a number of successive stages (the forming of breasts, the first menses, the beginning of sexual activity, …), and the longer time of generational renewal, marked by the possibility of motherhood (Maciejewska-Mroczek et al., 2019). For women who suffer from this genetic condition, the gap between the physical development in time and social status associated with age, gender and generation, may engender a persistent feeling of “being out of place.” The exceptions due to this chromosome disorder allow us to demonstrate the importance of the “right” time for the fabrication of a “right” body, as well as the power of the norms that connect bodily transformations to the age and thresholds fixed by society. As the term “age” is polysemous, we will distinguish throughout the article between chronological age, age status, the stage in life, and age linked to a generational position. These different meanings intertwine and overlap, but as they interact, they refer to various ways of finding one's place in a time disrupted by illness.

After presenting the research and the fieldwork, the article explores how measuring the body has become central in the social construction of age, and in the formation of the concept of “age-appropriateness” (Kelle, 2010). Then, we analyze how, among women suffering from Turner syndrome, the impossibility of conforming to such standards can produce a lack of legitimacy associated to various forms of desynchronization: between physical appearance, chronological age and age status; between the physical developments induced by hormone therapy and a particular stage in life; between chronological and reproductive age and generational position. Finally, the paper stresses how the relationship between body and time results from a complex interplay of social markers of age and gender that represent different ways of coping with the social and biographical situation.

## Fieldwork and methods

The present article is the result of an ongoing research project on the bodily experience and life trajectories of people suffering from Turner syndrome. This is a rare genetic condition, due to complete or partial absence of the second chromosome X, that affects about ½, 500 newborn females in France every year. The consequences are small stature, ovarian insufficiency resulting, for the most part, in a delay or absence of pubertal development, and infertility. Morphological specificities and associated disabilities (e.g., hearing deficiencies) as well as an increased risk of diseases caught later on can also appear. Hormone treatments—both growth and sexual hormones—can be part of the treatment. Such pharmaceutical corrections are part of a molecular fabrication of age and gender (Gaudillière, 2003; Murano, 2019) that calls into question how the medical profession, as well as women and their entourage, deal with the risks of normalizing the body by its “enhancement” (William et al., 2011; Rajtar, 2019). The complex history of Turner syndrome also gives an insight into the scientific debate on sex/gender variations, which is “good to think with” as Löwy recalls quoting Lévi-Strauss (Löwy, 2019: p. 31).

The fieldwork that gave rise to this article was conducted in France and carried out through ethnographic and participative methods. We met 20 girls and women between the ages of 10–60, all heterosexual, from different social classes and with varying educational backgrounds. Skin color, migrational background or the experience of being racialized or ethnicized are not significant in the population interviewed. One person had recently migrated from Central Africa to join her sister in France and to receive medical follow-up.^4^

The first interviewees were approached through a message that circulated thanks to a not-for-profit organization, explaining the objectives and methods of the research. After the initial contacts, people were recruited through a snowball effect. We met the respondents in their own homes on several occasions, some of them twice, and sometimes in the context of the activities of a patients' association. This approach allowed us to gather narratives in a variety of speaking situations: formal interviews, informal exchanges, for example at lunch or over a coffee, and conversations between women or girls affected by the syndrome. The formal interview guide was organized around six topics: the discovery of TS and the care trajectory; the experience of growing up and aging; the age transitions and the expected physical transformations; the evolution of the relationship with one's body, with oneself and with others; the competences engendered by Turner's syndrome; the perspective on both medical and associative care. As is common during ethnographic fieldwork, other issues arose during the discussions. In the case of the younger participants, we also met their parents and sometimes their siblings. During 2 years, we participated in the activities of a patients' organization.^5^ We also held two workshops on what it means to grow up with Turner syndrome. We then carried out a content analysis, paying particular attention to contextualizing the narratives and pointing out the discrepancies and convergences between the different issues with respect to the modes and contexts of the data collection (e.g., formal interviews or observation).

The narratives and experiences are extremely diverse. Working with a group suffering from a rare syndrome poses the problem of constituting a population that has homogeneous social characteristics, while the women we worked with have different ages, family and professional situations and largely different histories with the disease. This diversity makes it difficult to interpret the influence of class and “race” on women's experiences. On the other hand, it has enabled us to highlight the importance of age, gender and generational positions, which emerge as transversal elements in the construction of a legitimate body. The variability is also linked to the specificity of syndromes, which are not diseases and manifest themselves rather like the expression of an anomaly (Canguilhem, 1943). A syndrome can vary greatly in shape from one person to another and its manifestations will unfold along widely diverging timelines.

Finally, there is the history of Turner syndrome itself, starting with its first description dating back to 1938. Its chromosomic origins were identified in 1965 and growth and sexual hormone therapies, were widely adopted only from the 1990s onwards. Today the diffusion of prenatal screening has contributed to parents being informed at an increasingly early stage, which allows them the possibility to interrupt a pregnancy or anticipate the hormone treatments for their little girl. Treatments have evolved too, having become more individualized with a more refined adjustment of dosages. Because of this great diversity, we have decided to present our findings under four narratives. They echo the testimonies of several of the women we met, while allowing for a better contextualization of their statements and experiences. These four cases were selected for several reasons. First, each of them is particularly representative of the form of desynchronization that we wish to highlight. Second, the chosen narratives are particularly dense with information that makes it possible to situate the experience of Turner syndrome as disorder in time and in status in the overall life course. Third, the four individuals are very different with respect to social class, family history, level of education, and place of residence. The narrative of the first interviewee is characterized by the durable experience of poverty and social marginalization in a small provincial town. The second interlocutor belongs to the urban wealthy bourgeoisie, and has been educated in some of the best schools in the country. The third person has experienced a history of upward social mobility, as she is the daughter of farmers who ended up in a recognized and well-paid profession. The fourth subject belongs to the middle class, characterized however by a significant intellectual “capital” in Bourdieusian terms. The choice of these narratives is not meant to associate social profiles with experiences of the syndrome. However, we considered it important not to eliminate the diversity of social conditions and to situate the narratives in specific contexts.

## The normative power of physical development

The need to organize bodily changes and to establish fixed thresholds for different ages took on a particular dimension between the 19th and 20th centuries. Scientific measuring of the body accompanies child policies, as well as policies concerning adolescence and old age. The techniques of surveillance medicine such as screening, population studies, statistical enquiries, and public health campaigns have turned the variations of these changing bodies into measurable, understandable, objective, and predictable phenomena (Armstrong, 1995). Developmental thinking has introduced the idea of a life cycle divided in a regular and universal succession of stages (Turmel, 2008; Diasio, 2019b), which relating bodily transformations to a specific vision of time, seen as linear, irreversible, progressive and teleological. This epistemology and the apparatus it deploys aim to stabilize the mutability of the body. The purpose is to distinguish between changes that happen over the course of a life and are associated with a social age, and the modifications that stem from a changed state of health. Science has taken up the challenge of making these variations measurable, understandable, objectifiable and predictable, particularly in fields like pediatrics, psychiatrics and geriatrics. This process has contributed to a conceptualization of the “healthy” body as a “stable” body (Armstrong, 1983; De Swaan, 1990), and of adulthood as the age of stability.

The variations also define which dispositions and behaviors are possible, or even recommended, for which times in life: psycho-cognitive development tests for children or graphs measuring the autonomy of older people are examples of this. This means that age-appropriateness, now more than ever, constitutes a central component of the management of people's existence: the beginning of school-going, leisure and sports activities, relationships and sex lives, and ages to become a parent (or not) and to leave one's “active” life. The physical, cognitive or psychological measures intertwine with political decisions in order to find the best regulation of people's life courses.

The association of age categories with the social distribution of competences is not a prerogative of so-called Western modernity, and several societies institute “a normative relation between a certain age and an activity” (Widmer, 1983: p. 346). However, in the technology-based contemporary societies, this combination is founded on the scientific measures and its legitimacy is reinforced by a linear, chronological and mathematical definition of age. The contemporary fascination for quantification (Voléry, 2020) also combines with the standardization, the bureaucratization and the individualization of age as “a neutral and universal criterion for public action” (Rennes, 2019: p. 266). These quantified measures are appropriated by the social actors as a way of precisely establishing the stages of their development: children, who are the first target of this process, do not hesitate to declare, for instance, that they are “8 years and 3 months” old. The bodily changes and the measure of age are thus at the very core of their social identities (James, 1993).

The importance of measuring has two consequences. Firstly, it reinforces the effects of the naturalization of age and the difficulty of thinking this dimension as a social category with its effects on the people being categorized (Hacking, 1999). The government of time and of the body's instability is a central dimension of a biopolitical order, but it is a “soft” biopolitics (Diasio, 2019a), all the more efficient because it is so obvious. Secondly, the concern with defining more and more precisely what is “age-appropriate” opens up areas of uncertainty (Kelle, 2010). The more we try to grasp universal criteria of development, the more we encounter variations, nuances and idiosyncrasies. That is how diagnoses of non-conformity with age-appropriate-development have also become so widespread in the field of formal school learning, and as in the alarm about the precocious puberty in spite of the controversial medical data (Cozzi and Vinel, 2015; Piccand, 2015). The medicalization of ages that are judged to be “critical” (e.g., puberty, menopause) entails both an eagerness to define what is proper to a given age, and the difficulty to distinguish between “normal” and “pathological” changes. “The body's aging process, whether in childhood or in later life, has become, in itself, problematic during the course of the 20th century in Western societies (…) the instability of the aging body, coupled with a decline in childhood mortality and increasing life expectancy, has worked to blur biomedicine's normal division between natural and pathological bodily change. And, in so doing, it has produced a range of new uncertainties about the life course as lived” (James and Hockey, 2007: p. 143). Finally, the measuring of ages and bodies is now faced with a medical world that has become more and more individualized and where the molecularization and singularization of treatments call into question instituted categories (Rabinow and Rose, 2006; Raman and Tutton, 2010).

An illness or a genetic condition will deeply affect this normalization of the link between body and time. A serious child disease will place the child in a position that may put it “outside of childhood” (Bluebond-Langner, 1996), while older people faced with degenerative processes are described as returning to childhood (Hareven, 1995). In other circumstances, such as with the case of myotonic dystrophy, adults who are considered at the height of their strength will have to take the same precautions as older people (Perrot, 2021). Such imbalances do not necessarily lead in the direction of increased fragility: children suffering from type 1 diabetes can outperform adults when it comes to auto-administering their care (setting up the catheter, injecting themselves), by subverting attitudes that are usually associated with their age and, sometimes, their gender (Williams, 2000; Renedo et al., 2020).

With Turner syndrome, the experience of stunted growth, small stature and late puberty highlights the normative power of the “right” kind of development when it comes to defining who one is and what one's place is: “A friend of the family described me as an old child, and I proudly compared myself to Peter Pan. […] I might not object to womanhood, but I could not imagine myself as a woman. Being a kid defined who I was” (Beit-Aharon, 2013). This temporal disorientation can also translate into a confusion about one's status: am I a child? An adult? A woman? We will focus now on four “cases” that exemplify, each one in its peculiar way, the diversity of misalignments between body, time and gender norms. These stories also present women of different ages, social conditions and family histories. As such, they require more in-depth exploration to set their experiences in perspective and in a specific context.

## Nadine: “As a child treated worse than dirt”

Nadine is 55 and was born and raised in the East of France. She lives alone in social housing, in a suburb of a median size town. Her apartment carries the traces of a medicalized life: her bed is equipped to facilitate her weekly injections, there are piles of drugs in the bathroom and living room, and she has a regulator for the sound on her TV to help her with her hearing problems. As she is currently working as a home care assistant, she could be described as the help who “does the dead,” as Verdier (1979) put it. That is also how she talks about her work: “*I close their eyes,”* she says, and proudly recounts how she was able to accompany several people all the way to the end of their lives.

As the eighth of eleven siblings, born to a family of farmers, she was raised by her grandmother until she was 11. When she was a little girl, she was already “*always*” getting sick. Her frail health and her difficulties at school made her father decide to make her abandon school. That was her first struggle, because she enjoyed studying, even though she was slow and had to suffer the mockeries of her classmates who called her “the dwarf.” Then, when she turned 14, she left school and entered an institution. Fourteen was also the age at which her parents, worried about the fact that she still had not had her period, took her to a doctor: “*They took me to Paris, I underwent some tests and all that and that's where they discovered I had Turner syndrome. But nobody told me anything. When I was at school […] it was the nurse who would give me the drugs that made me have my period like everyone else*.” The nurse came to the refectory every day at 12 to distribute the boarders' medication. Nadine would remain in the dark about her illness until she turned 24: “*By dint of … my parents ended up telling me. And then after a while I also asked the doctors*.” She received very vague explanations about some genetic problem, until the day she obtained more precise information through a patients' organization.

This struggle for knowledge was doubled by a permanent struggle for recognition of herself and her abilities, in spite of her small stature. “*I was treated like dirt*,” she often repeats. It was a struggle against her parents who considered her “*inferior*” to other children, a struggle for the right to get her driver's license and her first responder's certificate, or her diploma of home care assistant. As she says: “*OK, we're not tall, but I do the work just as well as a person of natural height*.”

In her account, her small stature appears to be the source of a double process of inferiorization and invisibilization. It puts her in a status of “a child,” and “a sick child” at that. “*To make people see that I'm here, that I'm fighting, that is very hard […] when I go [to the doctor] with someone, he will be talking to the tall person who accompanies me, instead of talking to me*.” The struggle to be seen and heard influences the affirmation of her femininity too. Being a woman with Turner syndrome means “*fighting, thinking, and making oneself heard*.”

Nadine is thus clearing a path for herself where she can be seen and heard. She loves music, and at one moment, during the interview, she sings in a confident, well-placed, and resounding voice. She sings at family gatherings or with friends, dressed as a boy, and incarnates male stars of French pop music from the seventies. Several of their portraits adorn the walls of her apartment. As she is squeezed into the position of a child, which contrasts with her chronological age, Nadine mobilizes her body to “*show herself* ” and “*be heard*,” and her entire account is structured on these different sensory matrixes.

## Corinne: Going through life with a persistent feeling of illegitimacy

Corinne is a 60-year-old biologist who works in the agro-food industry, is married and has adopted a child who is now 15 years old. The family lives in an individual house with a small garden, in a suburban town in the Southeast of France. She comes from an upper-class family with a father working in the industrial sector, a mother who was a homemaker and a younger brother, making up a family marked by the silence surrounding her “illness.”

After “*having been stretched out in every direction*” to trigger her growth, the Turner syndrome diagnosis was given when she was 14, by a renowned Parisian doctor who did not deem it necessary to inform the young patient about her condition. However, her mother would immediately tell her, since, Corinne being an intellectually precocious child, she was about to pass her baccalaureate at age 15. This institutional threshold therefore influenced her illness trajectory (Corbin and Strauss, 1988). At the time, all the information she received centered on the absence of fertility: “*no growth, no puberty, you won't have children*.” Corinne discusses the violence of the diagnosis, and how her mother's view of her would from then on transform her into an “*a-sexual being*” trapped in the generational position of the “girl” who had no right to a sex-life, love, or motherhood.

The hormone treatments, which were still in their early days in the 1970s, changed her body: she gained weight, and the presence of male hormones preoccupied her*:* “*what am I going to turn into?*” The stages in the treatment and her own transformations are both discussed in terms of the chemical products that acted on “*[her] fabricated body*”*:* the nandrolone phase, the trophobolene phase, and the estradiol-progesteron phrase. Her “*accelerated puberty*” was overwhelming and depressing. Biological and social stages succeeded one another—puberty, college, first job, very tardy first amorous relationships, adoptive motherhood—but she felt out of sync and often saw “*an overcast horizon*.” Being out of step with time, and having her intellectual qualities dissociated from her childlike appearance, contributed to amplifying the feeling of not “being in her place,” which constitutes the red thread of her narrative.

“*Considering that I was intellectually precocious and at the same time I was physically not developing, I cannot tell you how hard it was to deal with that gap. I was at university with people who were 19–20, had no breasts, and I was 1.35 meters tall. How do you find your place in that? How do you find your place among them? Some of them were already in a couple, anyway (knocks on the table). You ask yourself: what am I doing here?*”

In her testimony, her small stature is constantly tied to a femininity that is perceived to be defective (often expressed through references to the lack of breasts) and a sexuality that does not follow the same stages as the others: her very late first kiss, and her stormy sentimental relationships. The fact that physical development, belonging to an age and gender, and being in the “right” stage of life are all out of sync, manifests itself in a variety of areas. When she gained a lot of weight due to a bad dosage of her treatment, her mother would dress her in pregnant women's clothes. At work, she is not taken seriously. When she decided to adopt, the employee turned to her husband to place the baby in his arms, which strengthened her feeling of illegitimacy: “*I felt illegitimate, completely (Pause). Because you are always asking yourself the question, whether you are legitimate. […] You never really feel completely legitimate, as a woman. I never feel completely legitimate as a woman*.” The illegitimate body is, in Corinne's experience, entangled in age and gender non-appropriateness. The gap between body and time is here described as a difficulty to settle in the conventional stages of life (Settersten, 2002). As in the case for Nadine, it is possible to find a place at the margin or to occupy a void. That way, with friends, “*we have a specific place, one that does not belong to anyone else. […] We are not a threat, neither for the boys nor for the girls. We're the good friends, we make everyone laugh, we accommodate everyone, we smile all the time and accept everything*.”

## On the other side of reproductive life: Emilie and the menopausal turn-off

These cases can be associated with a time of late diagnoses and limited treatments. However, the feeling of illegitimacy caused by a body that does not show the “right” markers of gender and age is still just as pervasive among our younger respondents. Moreover, being assigned to childhood is then doubled by another positioning “outside of any age,” namely menopause. During one of the first meetings we attended between Turner syndrome women and girls and gynecologists, one of the criticisms patients voiced was that the hormone substitution treatment mentions “*women who could be 51*,” and this is “*very problematic*.” This discussion, though it seemed banal at the time, would subsequently become quite meaningful.

Emilie is a 36-year-old manager. She lives in a big city in the West of France and is now going through some great existential changes: she has new professional responsibilities, and she has entered a relationship in which she lives with her partner as a couple, after a deferred love-life and a “*period of great mistrust toward men*.” Her parents were farmers, and they both suffered from other chronic illnesses themselves. She was diagnosed at age 14 because of slow growth and a lack of any signs of puberty: “*I never had let's say the physical passage … you progress in age, in life, but your body itself does not evolve, it is at a standstill*.” Between 1999 and 2019, the treatment of the syndrome happened haltingly, with many stops and starts, and periods of waiting. In 2019, Emilie says she felt “*ready without really being ready*” to follow a treatment and it was only gradually that “*in my head it had matured, well I'm soon going to be 35, I will have to do something. So I began the treatment in January 2020*.” This interior time was a long, personal and rugged road that clashed with the rapid physical changes induced by the first growth hormone cycle, which had made her grow over 20 cm in a very short time.

The fragmented way in which the treatment was then followed up was enhanced by the discovery that she suffers from epilepsy, which for a while relegated the syndrome to the background and with it, her relation to “*Emilie the woman*.” When she discusses this, she talks in the third person: “*that part of me that is the syndrome, that part of Emilie, but Emilie as a woman, not as a person, was beginning to fall asleep … and also the relation with men, and with her own body*.” The absence of a menstrual cycle, combined with a fragile bone structure and a disposition of the sexual organs that does not facilitate sexual relations, gave her the feeling that she had “*the status of a woman in her menopause*.” Emilie finds this idea “*embarrassing*” and “*psychologically complicated*” and associates it with the absence of a “*woman's life*”^6^: “*I did not have a woman's life as such … I had pushed it away somewhat, see? I was Emilie, a person, but I was not … I was not a woman* (She stops, tears in her eyes) *this is sort of the hardest part*.”

These words revive widespread social ideas that associate the end of the menstrual cycle with a *passage* [Skultans ((1970) 2007)] and the decline of femininity (Lock, 1993). The lack of the life of a woman is here translated too into a difficulty to approach sexuality, and the grief over the loss of maternity and a feeling of being “*mismatched*” and “*out of place*.” Family gatherings or reunions with friends strengthen the feeling of being “*the weak link, the ugly little duckling, the odd number*.”

Evoking menopause thus also stirs up another question, which is the one about filiation and one's genealogical position. The menopause for mothers and the first period for girls constitute a focal transmission point in contemporary France (Vinel, 2008). In many societies, the end of fertility means handing over one's legitimacy to procreate, even if that sometimes means, at least formally, to stop having sexual relations (Beyene, 1986; Delanoë, 2006). These rules, which can be transgressed through more or less explicit practices, aim to dissipate any possible confusion or ambiguity in the succession of generations. Mobilizing the image of the menopausal woman thus highlights another question: that of one's “place” in the order of generations, and of the contribution of women to the process of succession.

## Françoise, or the trouble in kinship

The inability to have children has serious consequences for the family group, because the transfer of reproductive power constitutes a structuring element of generational succession. The infertility linked to the total absence of X chromosomes is not an individual question: it also puts the continuity of the lineage at risk. As Radkowska-Walkowicz and Maciejewska-Mroczek (2023: p. 6) pointed out with regard to Poland, mothers whose children have Turner syndrome see their daughter's infertility as “as an interruption of the intergenerational transfer of norms and values [that] jeopardizes women's hope for future grand-motherhood”. That makes it one of the consequences of Turner syndrome that are the most difficult to deal with within the family. The silence around what is often an open secret goes beyond differences linked to age, different diagnoses, or therapeutic and biographical trajectories. Indeed, the silence surrounding infertility feeds into and is fed by a concern around the ability to procreate that also applies to other members of the family, among whom it induces a desire to investigate their own chromosome types, or a fear of maternity-linked events like pregnancy or late periods.

Relationships among siblings seem to be particularly troubled by sterility. The infertility of one is seen as a potential threat to the fertility of others. Moreover, because of the confusion between genetic and hereditary illnesses, the children of a brother or a sister can also be preoccupied that their aunt's sterility could spill over onto them. Lastly, the pregnancy of a sister, or the new fatherhood of a brother can bring about a lack of equality of place, and an asymmetry in generational ranking, which the women we interviewed experience with much apprehension.

Françoise is 50; she has an intellectually oriented profession and lives in the south of France. She is very active in the local branch of a not-for-profit, she welcomes us, puts us into contact with people likely to be of value to the enquiry, and encourages us to pursue. As opposed to the many silences that have parsed our fieldwork, she underlines the importance of “*talking, talking, talking*” about it. Psychoanalysis helped her to become aware of the questions of sexuality, to which she returns often during our meetings and which just as often are avoided in collective discussions. “*The question of infertility is a major worry for us; as is the question of sexuality, but people don't talk about it. When you have learned to dissociate womanhood from maternity, and maternity from sexuality, you are OK. […] But, you don't have tits you don't get a guy; and to have words to explain all this, that helps*.”

Her experience of “being small” is told with a mixture of tenderness and concern. She is tender when she evokes her relationship with her brother when they were children: “*He was very protective of me and his friends too, I was small, they never heckled me or pushed me around, they were very brotherly and adorable*.” However, she was impatient, “*worrying about a body that wouldn't grow*” and this becomes clear from a dialogue with her youngest sister during a workshop the patients' organization:

*Françoise: Marie-Laure had her period, and I was expecting to be next, my sister had gotten ahead of me*.

*Marie-Laure: we called her the* “*munchkin*” *(la puce)*.

*Françoise: yes, I had a grandma who was small*.

*Marie-Laure: Françoise didn't see anything coming, and I thought, lucky her*.

*Françoise: and I would cry […] At the time of the diagnosis, we knew something was not right with me, but until then I had my place among the siblings*.

*Marie-Laure: and then I became a mom, in 1980 we did not know that it was genetic. Moreover, in all this I was worried about her, about my big sister*.

“Finding one's place” by comparing the changes of one's body to those that are happening to other family members, especially those of the same sex, constitutes one of the ways in which people take up their place in a family (Diasio, 2014). Turner syndrome, however, turns these relations upside down. The youngest sister's first period had an impact on Françoise's status as the oldest daughter. The birth order and its connection to gender are fundamental in the relationship between siblings, even in European societies where the prevailing social norm is to consider siblings as equal (Segalen and Ravis-Giordani, 1994; Fine, 2011). Françoise's infertility subsequently comes to blur her place in the family. Nevertheless, the birth of nieces and nephews is told with much humor as a way of settling back into a genealogical order and compensating for the lack of motherhood without suffering its burdens.

## The complex interplay of bodily markers

In the experience of Nadine, the gap between chronological and age status, her small size and the childlike body discredit her and make her invisible as an adult woman. Corinne discusses how important it is to settle at the right moment of one's life course, in order to avoid a lack of legitimacy as a female adult, as a professional, and as a mother. Emilie's experience shows a tension between her life as a young woman and her menopausal status. The infertility and chaotic development of Françoise bring about disorder among the siblings and question her place in the generational rank. The four narratives presented here show how the relationship between body and time is not a well-oiled mechanism of biological data and social roles. They also reveal how the significant markers for the adjustment or misadjustment between body and time vary according to the moment of the life course, the social interactions and the particular temporality that is at stake.

The choice of age and gender markers is far from arbitrary or coincidental. As women with Turner syndrome deal with the numerous ways in which the social making of age and gender is expressed: in their accounts, the height, the breast, the infertility, and the absence of menses are the most relevant phenomena. These, however, may be differently stressed according to the biographical and social context of the women's experience, and to the tactics (de Certeau, 1980) they deploy to “make do” with this genetic condition. Height is certainly the body feature that comes up most frequently. It is mentioned in relation to the altered rhythm of individual growth, it reveals the presence of the syndrome, and it underlines the difficulty of being part of a life stage, particularly in relation to peers. The misalignment between height and age thus refers to different temporalities: that of growing up, of the illness trajectory, and of the succession of age stages.

However, stature materializes social, family, emotional and sexual relationships too. Height constitutes one of the first indicators brought to bear in measuring growth (Tersigni, 2015), but it is also a primordial expression of sexual dimorphism. It is the result of several genetic variants and social practices, such as unequal access to food resources (Guillaumin, 1992) or matrimonial choices, which have had an impact on genomes by selecting tall men and small women (Touraille, 2008). Height therefore constitutes a double operator in classifications of both gender and age. It indicates childhood, but also the fact of belonging to the female gender. In the case of women and girls with Turner syndrome, the question of height is ambivalent. Many of our respondents told us how their own mothers or other female members of the family are “small” and that this resemblance delayed diagnosis. The problem of height seems to become more acute when the child leaves the family circle, for instance at middle school, in their professional life, or in public spaces. In these situations, height is an element that materializes the disconnection between age and the fact of being hemmed into the children's category. As the word “small” signifies this double assignation, both to a physical dimension and to a state of social and psychological immaturity, the short stature embodies the asymmetrical and hierarchical relationship between adults and children. However, when these women evoke their love heterosexual relationships or interactions with male members of family (such as brothers or cousins), the fact of “being small” is less likely to be a source of discrimination. The “male taller norm” that dictates that men should be taller than women [Bozon, (1991) 2006] is still important in matrimonial arrangements and constitutes a materialization of the gender hierarchy. That is why in a group interview we saw smiles and laughter of agreement when one of our respondents said, “*We Turner girls always go out with tall men!*” A gender perspective then subverts the stigma of an “age-non appropriate” height. Therefore, in some narratives, the small stature is considered as part of the “normality” of gender-based dimorphisms, which is in turn reinforced by the “normality” of heterosexual relationships.

This legitimization of small stature through heterosexual relationships may explain why the question of height comes up more painfully in the narrative of Nadine. She refers half-heartedly to her long condition of loneliness, repeatedly dodging the question of sexuality and love relationships, and justifying her single state by referring to infertility. Françoise's attitude, on the other hand, is different: in her account, the short stature is the trigger for the diagnosis, the proof that “something is going wrong,” but it also gives rise to a protective attitude on the part of her brother, which is recalled with tenderness. In her account, especially when she talks about her heterosexual relationships, infertility plays the main role. Infertility also gave rise to her psychoanalytical treatment in order to learn to dissociate femininity from motherhood. Should we see this difference as an effect of social class and level of education between two persons with very different social positions? Maybe, even if our data do not allow us to make definitive interpretations.

The absence of breasts and infertility are particularly emphasized in sexual and love affairs, in interactions with friends, especially in youth transitions that put gender identity at stake (Fingerson, 2006), or in family relationships that involve intergenerational transmission. Nevertheless, while menstrual blood is often socially considered as a gendered matrix of experience, which permits “a shared subjectivity” (Pandolfi, 1991: p. 155) and a bodily mapping of gender difference (Prendergast, 2000), the girls and women we met undermine the importance of this fuzzy web. In fact, the attitude toward menstruation is closer than one might imagine to that of French adolescents encountered in other research (Mardon, 2009). The menarche is part of the definition of growing up and indicates a good state of health, nevertheless the presence of first menses is not a sufficient condition to become “women” (Diasio, 2014). However, the importance given to periods depends, more than other factors, on the moment in which these women and girls are interviewed. In adolescence, the onset of menarche is considered rather as a way of aligning with the experience of other girls and finding one's place among peers or in the family, whereas over time the presence of monthly menstrual blood may be considered as an unnecessary bother. However, menstruation may regain relevance in the context of a relationship with a male partner who may regard it “as a sign of womanhood”, as a 40 years old woman says.

The approach to menstruation also depends on the generation to which the girls and women interviewed belong. For Corinne, who was treated in the 1980s with hormone therapies that were still in their trial and error stages, the onset of her first period came late and followed periods of “self-manipulation,” as she calls them, which were particularly painful. For Nadine, the menarche was experienced in misunderstanding and the passivity of a pill silently swallowed in the school canteen. These experiences invite us to situate the construction of a legitimate body in another temporality, which is the history of the discovery of the syndrome, its care and the evolution of treatments. A whole history of the patient and his or her participation in care is also intertwined with these transformations. Thus, the youngest girls in our population live in an age in which a new vision of children as present beings (Lee, 2001) leads doctors and parents to encourage their participation in therapeutic choices.^7^ Children and teens can then be consulted to know if and when to initiate treatment with sex hormones and to dissociate, for example, the growth of the breasts from the onset of menstruation.

Physical transformations that happen over time are thus a part of a continuous and multidimensional process of biological and social facts which is open to interpretation, appropriation, and even conflict: “Over time, the combination can at times be harmonious, and at other times dissonant, and the individual is confronted with contradictory injunctions and “moral tensions” (Peatrick, 2003: p. 16). In the biological continuum, some markers will be socially selected (or not), physical qualities will be encouraged (or not), and certain practices valued, while others will be left by the wayside. We can therefore adopt, for age, the same statements that feminist biologist Fausto-Sterling (2000) dedicates to gender: “Our bodies are too complex to provide clear-cut answers about sexual difference. The more we look for a simple, physical basis for ‘sex', the more it becomes clear that ‘sex' is not a pure, physical category. What bodily signals and functions we define as male or female are already entangled in our ideas about gender.”

## Out of the obvious: The body against “nature”

While the syndrome causes distress and a sense of lack of legitimacy, it also gives rise to another form of “presence in the world” (De Martino, 1948) that leads to a critical re-examination of hegemonic models of womanhood and their intersection with life stages. Radkowska-Walkowicz writes of an “emancipatory model of femininity” (Radkowska-Walkowicz, 2019: p. 138). Instead, we noted a reflexive stance: being aware of the normative power that the measurement of bodies obtained in contemporary society, the women and girls we met assiduously exert a distanced and critical gaze on their life path and the intertwining of bodily manifestations, age and gender positions.^8^ From one account to another, we find recurrent statements: “*Turner girls think a lot*,” “*We have to think twice*,” “*We think more than those who have no disability*.” Thus, Corinne claims that the syndrome brings “*a different view of femininity, and of men. […] We think a lot about sexuality, about…well, about many things, about the couple, about the family, we think a lot. And young girls who have nothing [i.e., who have no syndrome], who haven't thought about it at all, who throw themselves into their life, their love life without having thought about it for a moment, well they don't ask themselves:* “*can something else exist, can we get there in another way? So they put on make-up, they flirt, they appeal and that's it*.” In these words, it is less a matter of distancing themselves from forms of coquetry or so-called “feminine” aesthetic practices than of questioning their obviousness.^9^

Even if the experiences and illness trajectories are heterogeneous, we can observe the practice of a “bioreflexivity” (Memmi, 2003), which encourages women, at different moments of their existence, to question the influence of the syndrome on bodily states intertwined with age status and gender role. The stages of existence, which seem to occur “naturally” for those not affected by the syndrome, are submitted to a relentless evaluation to understand “*how one stage succeeds another*.” The body, its changes over time, and in particular its gendered expressions, such as the presence of menstruation or the development of breasts, are often described as “*artificial*,” “*manipulated*,” “*weird*,” “*fake*.” This lack of “*naturality*” flushes out the apparent correspondence between biological and social age and raises questions to understand “*where we stand*.” As Maëlle (21, assistant nurse) says, “*growing up, becoming an adult, means growing in awareness of what Turner is, of what the relation to the syndrome is*.”

It is interesting to note that in the discourse the term “phase” is frequently used in place of the “age.” The phase eludes naturalization. It is constructed at the intersection of a type of treatment, such as the “nandrolone phase” mentioned by Corinne, a step in the care trajectory, an experience of the body, such as a slowed or accelerated stature growth, and an existential bifurcation: leaving one's parents' home, a break-up in love, entering or quitting a professional activity. Ages and their thresholds are then thought otherwise than in reference to the apparent concordance of biological transformations and social positions. Thus, if being assimilated to a menopausal woman when one is young is rather uncomfortable, as we have seen with Emilie's narrative, the onset of aging and the cessation of hormone treatments are interpreted as an alignment with the experience of other women in their menopause. Far from challenging the sense of womanhood^10^ the moment of entering an artificial menopause thus constitutes a sort of return to the circle of so-called “normality.”

This process of demystification of the obvious leads our interviewees to unravel some dimensions of being a woman, which, from their point of view, are wrongly considered interdependent. “*The first thing people ask you when you talk about not being able to have children is if you have your period. It is weird, like it is a sign of fertility. That's why it's problematic […] you can have your period and be infertile […] for me it's decorrelated*” (Ariel, 40 years old, employee). As we have seen in Françoise's narrative, a cascade of decorrelations challenges common associations that women with Turner syndrome affirm enduring in everyday life: the association between chromosomal sex and femininity, between femininity and seduction, between femininity and maternity, between maternity and sexuality, or between the menstrual cycle and fertility. Furthermore, women with Turner syndrome deal with the numerous ways of conceptualization of sexual polymorphism (chromosomic, gonadic, hormonal, morphological…) and split up the variable expressions of gender. Throughout the interviews, the researcher witnesses the attempts to unfold, as much as possible, these gender attributions and overlaps that are perceived as problematic.

The questioning of the social and cultural evidence of “femininity” is often expressed through fighting metaphors. Becoming an adult woman is often associated with learning to fight, to struggle, to not be crushed, and above all to start talking, to no longer be suffocated by the blanket of silence in which one was enveloped (and cloaked) during childhood (Laiacona, 2019). Nadine's narrative is an example of this warrior attitude: “*I'm a fighter and I'm going to do everything I can to…to succeed if I fail. I am really a fighter. It is long, but I fight, I fight. That is what is good [in Turner syndrome], I try to show that I am here (she pounds) that I can*.” The words are often punctuated by gestures, such as the pounding of the fist on the table, which materialize these claims of fighting spirit. Becoming a woman means learning to “be seen” and “be heard,” where Turner's syndrome amplifies the invisibilization and minorization of women in relation to men, namely in the public space. Thus, one interviewee recalls how, faced with her repeated requests for a document to the administrative services, she would have had to ask her husband to intervene (which she did not) with “*his big size and his big voice […] Not only are we women, we are also small and childlike!*” This reflexive and fighting womanhood may constitute an example for other female relatives. The older women in our population point out how they establish special relationships with some nieces who turn to them for counsel or advice. They may be girls who have not yet had children, who are late in their first period, or who are complaining about family difficulties. These elective bonds reinstate our interviewees in a generational position.

Thus, the body, in its multiple expressions, may be a resource for resistance to these classifications, and to naturalization. For instance, while Nadine's height inferiorizes her, her voice, whether through singing or through her rants at physicians (“*I'm sure they heard me!*”), comes to her rescue to allow her to assert herself with family, friends, and medical or professional milieu. In Françoise's case, her small stature and gender asymmetry generate a protective attitude on the part of her brother, her brother's friends and in the close ties which she entertains with her nephews and nieces. This means that the experiences of women who have Turner syndrome demonstrate how “the body is not only shaped by social relations, but also enters into their construction as both a resource and a constraint” (Prout, 2000: p. 5). Taking this approach avoids the tendency to fall into the double reductionism of naturalization or radical constructivism, both in age (*ibidem*) and in gender (Touraille, 2011; Raz, 2019). It thus enables us to reconsider the “materialization of sex and the sexuation of matter” (Kraus, 2000: p. 190). While society acts upon the “unfinished body” (Schilling, 1993; Remotti, 2003) through cultural practices and a range of technologies of the self (Foucault, 1988), the body acts on society as a matrix of possibilities and limitations: its changes, its troubles, the play of its matters and contingencies, solicit choices and practices, and provoke social responses, power positions, and resistance.^11^

## Conclusion

To be an adult with a small stature and a childlike morphology, to become a “complete” woman at an inappropriate age, to go from late growth into an accelerated onset of puberty caused by hormone treatments: suffering from Turner syndrome brings about desynchronizations between several times. The temporality of syndrome, treatments, growth and aging, and filiation disrupted by partial or total infertility, are not coincidental, and the gaps between them may give rise to experiences of liminality (Turner, 1969: p. 95) and stigma.

The social norms that govern the “right” development of a body in time do not only establish appropriate age status. They are also at the heart of the ways in which gender is processed: it is not only a question of being at the right time, but of arriving there by negotiating the codes of a socially defined femininity. Lastly, the point is also to establish oneself in the long time of generations, whose renewal is threatened by infertility.

The troubled body is thus defined by a disorder in time, which is also a disorder in status that produces a lack of legitimacy in several areas: family, work, relations with medical staff, friends, or lovers. This discloses the link between body and time as one of the last bastions where social and individual existences are naturalized and essentialized. The experience of women having Turner syndrome also reveals the obvious, elusive, undiscussed character of adulthood, and partially of womanhood, in contemporary French society, and the silent force of the entanglement of age, gender, and generation in the course of the life.

Nevertheless, these narratives also reveal that in the thickness of the body and in its multiple manifestations, there are resources that go through the social evidence and put it into question. The body's materiality, with its complexity and singularity, plays against “nature”. The different markers used to denote age or gender only become meaningful and effective if they are situated in social situations and relations, including generational ones. For the girls and women we met, age and gender are not stable categories, they are rather forms of action that are expected in the context of a given relationship (Alès and Barraud, 2001): for example as daughter, sister, partner, aunt and so on.

Thus, day by day, with inventiveness, reflexivity, and humor, these women measure themselves to the established categories, find a place in their interstices, and continue to struggle, brave and pugnacious, for their own legitimacy.

## Data availability statement

The datasets presented in this article are not readily available in order to protect participant privacy. Requests to access the datasets should be directed to ND-nicoletta.diasio@misha.fr.

## Ethics statement

The research is registered with the CNIL data protection service of the University of Strasbourg at the following address: https://cil.unistra.fr/registre.html#proc-555. The CNIL is the National Commission on Informatics and Freedom (CNIL), which in turn is advised by the Advisory Committee on Information Processing in Health Research (CCTIRS). The studies involving human participants were reviewed and approved by Comite d'Ethique des Facultés de Médecine, d'Odontologie, de Pharmacie, des Ecoles d'Infirmières, de Kinésithérapie, de Maïeutique et des Hôpitaux CE-2022-137. Written informed consent to participate in this study was provided by the participants' legal guardian/next of kin.

## Author contributions

The author confirms being the sole contributor of this work and has approved it for publication.

## Footnotes

^1^Even though Marcel Mauss proposed, in 1934, to consider the body as a physio-psycho-sociological entity, for a long time anthropology and sociology, especially in France, have emphasized the discursive dimension of corporeality (Warnier, 2009).

^2^However, we do not underestimate these categories and their intersection in the making of body norms. For example, female adolescent bodies and their eroticization have been an historical, political and social construction in which gender, class and ethnicity assignments are intertwined (Walkerdine, 1998; Dorlin, 2006; Liotard and Jamain-Samson, 2011).

^3^We here consider generation as much a category that orders and structures society on the model of other social positions (Bühler-Niederberger, 2004), as a relational process through which family members are produced, legitimized or transformed (Alanen, 2010).

^4^In France, the term race is increasingly used among sociologists. In anthropology, however, there is an ongoing debate about the pertinence of this term, which fixes and essentializes a difference that is produced within asymmetrical social relations. The history of the discipline, the heavy heritage of physical anthropology, the fact that race does not constitute a category of public policy as it can be, for instance, in the USA, lead us to be cautious in using this term. I will therefore add inverted commas to this term or prefer the word “racization” which defines the way in which color, itself constructed, is part of a process of hierarchization of social groups.

^5^We cannot give more details about the places and organizations that were part of the fieldwork for reasons of confidentiality. The COVID 19 pandemic slowed down research between 2020 and 2021. All identifiable data that could identify the person are modified: first name, age, place of residence, profession. The first and last names are replaced by pseudonyms, as well as the place of residence and care by another city that comes close to the characteristics of the original one. The occupation is changed to another belonging to the same socio-professional category. These precautions are adopted in the case of all interviewees (parents, health professionals, etc.).

^6^Translator's note: In colloquial French, the expression *avoir une vie de femme* means to have a love life, and more specifically a sex life.

^7^Sometimes, the medical doctor invites the girl to observe at what time her classmates are menstruating in order to 'be ready' and to match pharmacological interventions to a form of social conformity (Laiacona, 2019).

^8^This concern with the normativity of body development also affects some parents of children with Turner syndrome, who question, for example, whether growth or sex hormones are necessary or not.

^9^Our first observations reveal that the approach to aesthetic codes also varies between generations. For example, a not-for-profit has recently set up workshops to encourage young girls not to neglect their appearance and to choose clothes that enhance their bodies despite their short stature. According to one of our eldest interviewees, there is a “*cultural change”* related to the earlier start of medical treatments.

^10^As demonstrated for instance by Delanoë (2006) in the case of France.

^11^However, we feel it is important not to idealize this combative stance, which we found in other experiences of chronic illness. The ideal of reflexivity and self-improvement may trap the patient in a model that he or she cannot achieve and bring new forms of determinism and disempowerment (Diasio, 2019a).
